# Critical Role of Constitutive Type I Interferon Response in Bronchial Epithelial Cell to Influenza Infection

**DOI:** 10.1371/journal.pone.0032947

**Published:** 2012-03-02

**Authors:** Alan C-Y. Hsu, Kristy Parsons, Ian Barr, Sue Lowther, Deborah Middleton, Philip M. Hansbro, Peter A. B. Wark

**Affiliations:** 1 Centre for Asthma and Respiratory Disease, The University of Newcastle, Newcastle, New South Wales, Australia; 2 World Health Organization Collaborating Centre for Reference and Research on Influenza, Melbourne, Victoria, Australia; 3 The Commonwealth Scientific and Industrial Research Organization (CSIRO) - Australian Animal Health Laboratory, Geelong, Victoria, Australia; 4 Department of Respiratory and Sleep Medicine, John Hunter Hospital, Newcastle, New South Wales, Australia; Kantonal Hospital St. Gallen, Switzerland

## Abstract

Innate antiviral responses in bronchial epithelial cells (BECs) provide the first line of defense against respiratory viral infection and the effectiveness of this response is critically dependent on the type I interferons (IFNs). However the importance of the antiviral responses in BECs during influenza infection is not well understood. We profiled the innate immune response to infection with H3N2 and H5N1 virus using Calu-3 cells and primary BECs to model proximal airway cells. The susceptibility of BECs to influenza infection was not solely dependent on the sialic acid-bearing glycoprotein, and antiviral responses that occurred after viral endocytosis was more important in limiting viral replication. The early antiviral response and apoptosis correlated with the ability to limit viral replication. Both viruses reduced RIG-I associated antiviral responses and subsequent induction of IFN-β. However it was found that there was constitutive release of IFN-β by BECs and this was critical in inducing late antiviral signaling via type I IFN receptors, and was crucial in limiting viral infection. This study characterizes anti-influenza virus responses in airway epithelial cells and shows that constitutive IFN-β release plays a more important role in initiating protective late IFN-stimulated responses during human influenza infection in bronchial epithelial cells.

## Introduction

The recent H1N1 influenza pandemic and the emergence of a highly pathogenic avian H5N1 influenza demonstrate the danger that this virus continues to pose, with its capability of evading our immune response and its ability to be rapidly transmitted throughout populations and across the globe. Much attention has focused on the ability of the virus to evade the host adaptive immune response through antigenic drift and antigenic shift of the virus and the implications that this has for the development of vaccines [1]. However, the ability of the virus to initially infect humans and evade early innate immune responses is less well defined, though is likely to be crucial in its ability to be transmitted and to cause disease.

Influenza first gains entry into humans via the airway epithelium yet little is known about this interaction and how it may vary between strains of influenza viruses, especially those that are more pathogenic to humans. As influenza viruses enter the airways the haemagglutinin (HA) glycoprotein on the virus attaches to airway epithelial cell surface glycoproteins terminating with specific configurations of sialic acid (SA) residues. Human influenza preferentially binds to SAα2,6Gal linkages that are predominantly found in the upper respiratory tract, while avian influenza viruses bind to the SAα2,3Gal residues in the lower airway [2]–[5].

The airway epithelium is an important contributor to the early innate immune response to virus infection. Type I interferons (IFN-α/β) and the recently discovered type III IFNs (IFN-λ1, -λ2, -λ3) are central players in innate antiviral responses, since IFNs initiate signalling cascades that lead to the containment of viral spread and subsequent activation of the adaptive immune response [6], [7]. Following successful entry into the cells influenza RNA is recognized by the intracellular RNA helicase retinoic acid-inducible gene–I (RIG-I), which leads to the production of type I and type III IFNs via transcription factors, interferon regulatory factor (IRF) 3 and IRF7 [8]–[10]. The released type I and type III IFNs then bind to their respective receptors IFNAR2 and IL-28Rα/IL-10Rβ on the same and/or neighbouring cells, and this initiates the expression of over 300 IFN-stimulated genes (ISGs) [11]. Many ISGs such as IFN-inducible protein kinase R (PKR) signals to degrade viral RNAs and also initiate apoptosis within the infected host cell, thereby limiting viral replication [12]–[16].

Infection with human influenza including H3N2 and H1N1 has been shown to up-regulate RIG-I, type I/III IFNs and various ISGs including PKR in dendritic cells (DCs) and airway epithelial cells [17]–[21]. While these studies demonstrated that DCs are the main producers of type I IFNs in response to infection, studies on the ability of BECs, which is the primary infection site that supports viral replication, to respond to influenza infection is limited. In addition numerous studies have shown that the highly pathogenic avian influenza H5N1 strain has a high mortality rate and have investigated the underlying reason for its high pathogenicity [22]–[25]. Nevertheless little is known about the kinetics and effectiveness of antiviral responses to influenza infection in primary BECs (pBECs) and how these may differ in response to different virus strains. We have previously shown that BECs mount an effective innate antiviral response mediated by RIG-I with the robust release of IFN-β, IFN-λ1 and ISGs to the low pathogenic avian influenza virus H11N9 [26]. In contrast this response was markedly impaired to a human influenza H3N2 virus. These different responses were the result of differential effects of viral non-structural protein 1 (NS1). Influenza viruses encode NS1 protein to inhibit host antiviral responses, and the NS1 from the pathogenic H3N2 strain more effectively inhibited RIG-I mediated signalling and the inductions of type I and III IFNs, whereas the NS1 from the H11N9 strain did not. Several studies have also shown that the NS1 protein of highly pathogenic avian H5N1 virus is also very potent in the host antiviral suppression [27]–[29]. Intriguingly, we also observed that IFN-β protein release occurred even in the absence of influenza infection [26]. If and how this constitutive release of IFN-β contributes to antiviral responses to influenza infection remains unknown.

In this study we assessed the antiviral responses of human BECs to human influenza, H3N2, and highly pathogenic avian influenza, H5N1, using a proximal airway epithelial cell line, Calu-3 cells, and pBECs obtained by bronchoscopy from healthy volunteers. We have defined the critical role of the constitutive IFN-β release during influenza infection, which is required to initiate the early induction of ISG expression and infected cell apoptosis. These factors are critical in the ability of BECs to limit influenza replication, even in the face of effective inhibition of inducible type I IFN responses by the influenza NS1 protein.

## Results

### Influenza viral replication in BECs is independent of sialic acid residue expression

**Figure 1:**
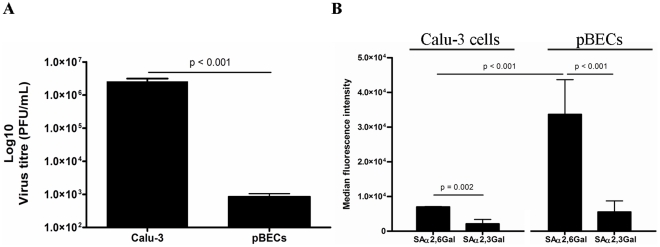
H3N2 influenza virus replication, SAα2,6Gal and SAα2,3Gal linked glycoprotein levels on Calu-3 cells and pBECs. Calu-3 cells and pBECs were infected with H3N2 influenza virus at an MOI of 5. (A) Viral replication was measured by plaque assay after 48 h. (B) The level of surface expression of SAα2,6Gal linked glycoproteins was measured by flow cytometry. Results were derived from three independent experiments and are presented as mean ± standard error of the mean (SEM).

We first compared the replication of H3N2 in an immortalized cell line (Calu-3 cells) and pBECs (Figure 1). A dose- and time-response of H3N2 was determined on both Calu-3 cells and pBECs with and without trypsin treatment, and MOI of 5 was shown to elicit appropriate induction of immune responses without causing significant cytopathic effect. Thus an MOI of 5 was used in all experiments. In these dose- and time-response studies trypsin treatment on Calu-3 cells and pBECs was found not to affect H3N2 replication rate and antiviral responses (data not shown) and was not included in subsequent experiments. After infection we found that H3N2 replicated to a significantly higher viral titre in Calu-3 cells compared to pBECs (Figure 1A). As influenza viruses bind to SAα2,6Gal residues to facilitate their entry into cells, we next assessed if these residues were more highly expressed in Calu-3 cells (Figure 1B). Surprisingly the opposite was the case, and SAα2,6Gal residues were expressed to a significantly lower level on Calu-3 cells compared to pBECs. This suggested that viral binding and entry into airway epithelial cells is not solely dependent on the level of SAα2,6Gal residues, and that post-endocytotic events are more important in limiting influenza infection.

### Antiviral responses in BECs are reduced during influenza infection

**Figure 2:**
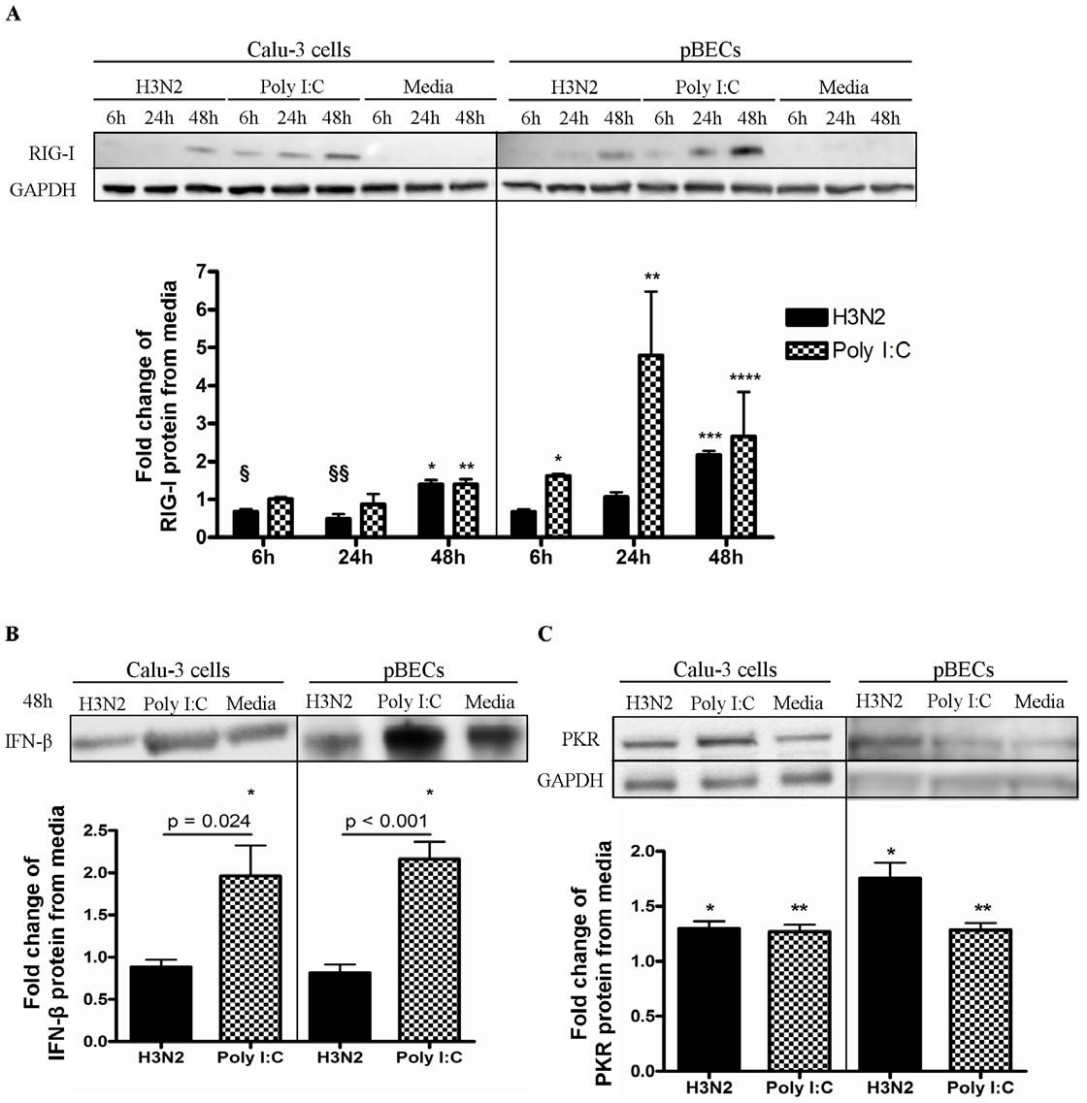
Type I IFN signalling and responses to H3N2 infection in Calu-3 cells and pBECs. Calu-3 cells and pBECs were either infected with H3N2 influenza virus or stimulated with Poly I:C, and RIG-I, IFN-β, and PKR protein production was measured by western blotting. (A) RIG-I in whole cell lysates compared to media control. (B) IFN-β in culture supernatants at 48 h compared to media control. (C) PKR protein in cell lysates compared to media control. Western blots of Calu-3 cells and pBECs were performed separately. Results were derived from three independent experiments and are presented as mean ± standard error of the mean (SEM). * - **** indicates significant induction from media control. § and §§ indicates a significant reduction compared to media control.

We then compared antiviral responses of Calu-3 cells and pBECs to H3N2 infection and Poly I:C, by assessing the induction of RIG-I, PKR and IFN-β genes and proteins (Figures 2, S1 and S2). H3N2 infection and Poly I:C stimulation resulted in significant inductions of antiviral genes in Calu-3 cells (Figure S1) and pBECs (Figure S2), compared to the media control. UV-inactivated virus was no different to the media control (data not shown).

Infection of Calu-3 cells resulted in an early induction of RIG-I mRNA at 6 h with a progressive rise (25 fold) to 72 h (Figure S1A). This was followed by increases in IFN-β (24–72 h) and PKR (48–72 h) mRNAs later in the time-course (Figure S1B–C). Infection resulted in the significant protein production of RIG-I (Figure 2A) and PKR (Figure 2C) but not IFN-β after 48 h (Figure 2B). Notably there was constitutive IFN-β protein present in Calu-3 cells cultured with media alone (Figure 2B).

In pBECs, infection induced an early and sustained induction of RIG-I mRNA (Figure S2A), this was again followed by a significant induction of IFN-β and PKR mRNA by 24 h (Figure S2B–C). Infection again induced significant protein production of RIG-I and PKR but not IFN-β after 48 h (Figure 2A–C). There was also constitutive IFN-β protein present in pBECs cultured with media alone (Figure 2B). Treatment of both cell types with Poly I:C resulted in the significant gene expression and protein production of all antiviral responses (Figures 2, S1 and S2).

These results indicate that during H3N2 infection of both cell types there was early RIG-I induction and later PKR production. In contrast although there was significant gene induction, IFN-β protein release was not enhanced.

### RIG-I induction does not affect influenza infection

**Figure 3:**
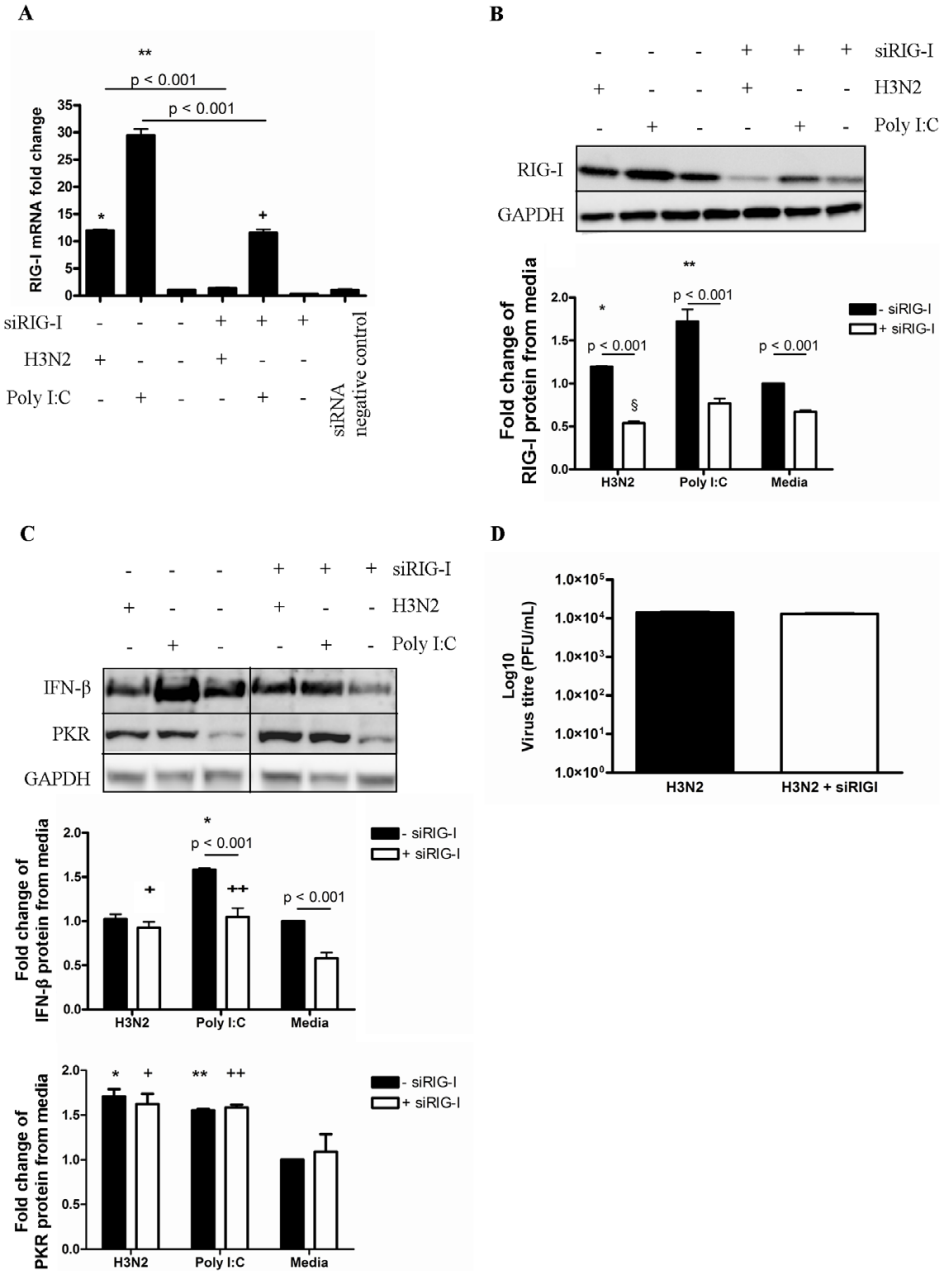
Role of RIG-I in IFN-β response to H3N2 influenza virus infection in pBECs. RIG-I siRNA was added to pBECs to silence RIG-I mRNA 24 hr before H3N2 influenza virus infection. Cells were then infected with H3N2 or treated with Poly I:C. (A) RIG-I mRNA was measured by RT-qPCR at 24 h. (B) RIG-I, (C) IFN-β and PKR protein was measured at 48 h by western blotting. siRNA negative control was also used to confirm RIG-I siRNA used was RIG-I-specific, and did not affect IFN-β and PKR protein induction (data not shown). IFN-β and PKR western blot was rearranged from the same blot. (D) Viral replication was measured by plaque assay after 48 h. Results were derived from three independent experiments and are presented as mean ± standard error of the mean (SEM). * - **** indicates significant induction from media control. + and ++ indicates significant induction from siRIG-I-treated media only control cells. § indicates a significant reduction compared to media control.

We then assessed whether RIG-I induction was important in the antiviral responses of pBECs against influenza infection. RIG-I was suppressed in pBECs using siRNA treatment before and during H3N2 infection or Poly I:C exposure and the effects on antiviral responses were assessed (Figure 3).

RIG-I silencing resulted in over 60% knockdown in RIG-I mRNA levels (Figure 3A). The use of a siRNA negative control was also used to confirm that knockdown was RIG-I-specific. In contrast to when RIG-I was present, when RIG-I was silenced H3N2 did not induce RIG-I mRNA and protein, and Poly I:C-induced RIG-I mRNA and protein production was attenuated compared to RIG-I-intact cells (Figure 3A and 3B).

When RIG-I was present IFN-β protein was significantly induced by Poly I:C but not by H3N2 infection compared to media only control (Figure 3C). When RIG-I was silenced IFN-β protein was decreased in RIG-I-silenced media control cells, however IFN-β protein was not reduced in H3N2 infected RIG-I silenced cells, and was significantly higher than that in RIG-I-silenced media controls. RIG-I knockdown partially reduced Poly I:C-induced IFN-β protein production, which was still increased compared to RIG-I-silenced media controls (Figure 3C). Critically the suppression of RIG-I also did not alter the replication efficiency of H3N2 influenza (Figure 3D).

Collectively, these results suggest that RIG-I is important in the induction of IFN-β expression, however it is not the only factor involved in the IFN-β response. Furthermore, low-level constitutive IFN-β may play a more important role in controlling H3N2 influenza infection than RIG-I-mediated inducible IFN-β response.

### Constitutive intracellular IFN-β is present and can be released during host protein synthesis inhibition

**Figure 4:**
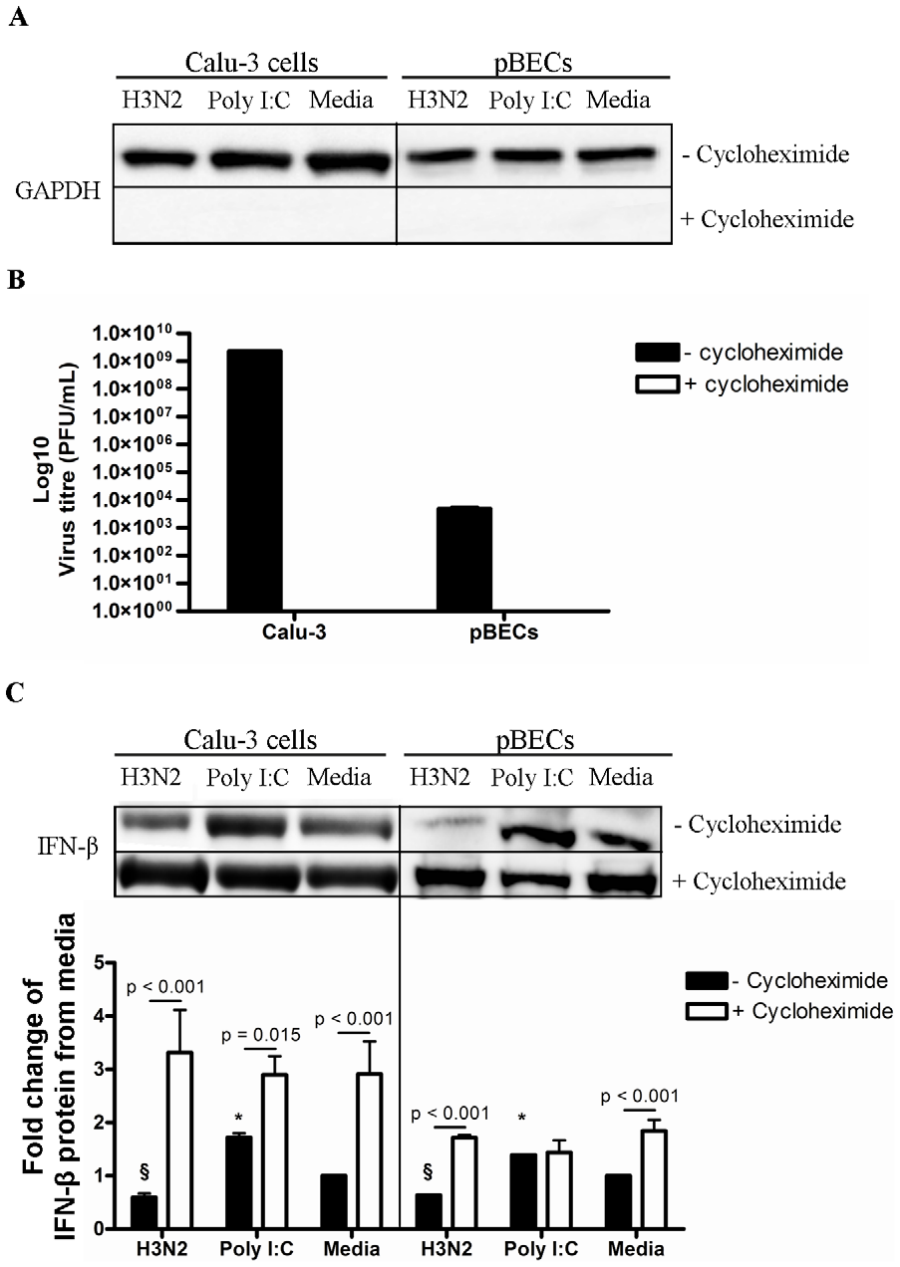
H3N2 replication and IFN-β release following protein synthesis inhibition with cycloheximide in Calu-3 cells and pBECs. Cycloheximide was added to Calu-3 cells and pBECs to inhibit protein synthesis 30 min before H3N2 infection or Poly I:C treatment. (A) GAPDH was measured at 6 h after H3N2 infection and Poly I:C stimulation by western blotting. (B) Viral replication was measured by plaque assay after 48 h. (C) IFN-β release was measured by western blotting at 48 h. Western blots of Calu-3 cells and pBECs were performed separately. Results were derived from three independent experiments and are presented as mean ± standard error of the mean (SEM). * indicates significant induction from media control. § indicates a significant reduction compared to media control.

We then further assessed the contribution of constitutive IFN-β protein production to antiviral responses against influenza infection. This was achieved by examining the effects of suppressing IFN-β production before infection (Figure 4).

Calu-3 cells and pBECs were pre-treated with cycloheximide to inhibit protein synthesis prior to infection. As expected GAPDH was not detected after treatment with cycloheximide, indicating successful inhibition of protein synthesis (Figure 4A). Cycloheximide also inhibited H3N2 replication by preventing host cell protein synthesis as expected (Figure 4B). Unexpectedly, however, there was an enhanced release of IFN-β protein in response to H3N2 infection, Poly I:C and media compared to that without cycloheximide treatment (Figure 4C). This suggested that the IFN-β protein released was pre-formed and stored inside the cell, and infection was triggering the release of pre-formed IFN-β.

**Figure 5:**
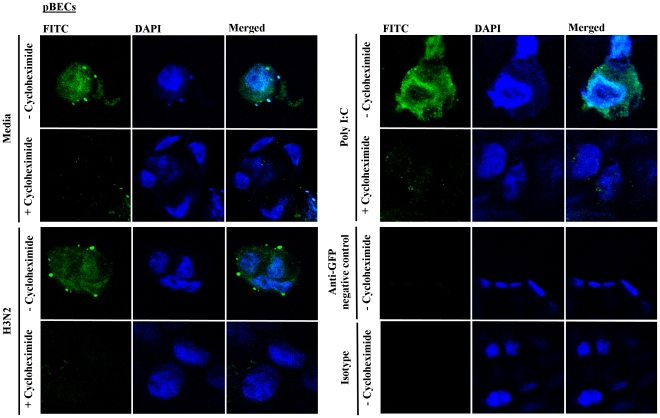
Intracellular release of IFN-β during H3N2 influenza virus infection in the presence of cycloheximide in Calu-3 cells and pBECs. Calu-3 cells and pBECs were pre-treated with cycloheximide and infected with H3N2 or treated with Poly I:C on coverslips and mounted on microscopic slides. The cells were then stained with goat raised anti-IFN-β antibody with secondary anti-goat IgG:FITC antibody. Goat raised anti-GFP antibody with anti-goat IgG:FITC and anti-goat IgG:FITC alone was used as negative control. The presence of IFN-β (FITC, green) was assessed by confocal microscopy. Nuclei were stained with DAPI. Goat raised anti-GFP antibody with anti-goat IgG:FITC, and IgG:FITC alone showed no non-specific binding in pBECs. Results were derived from three independent experiments.

We then assessed if pre-formed IFN-β was present intracellularly by staining pBECs 6 h after H3N2 infection or Poly I:C stimulation with and without cycloheximide treatment with goat-raised anti-IFN-β primary antibody and secondary anti-goat IgG:FITC antibody (Figure 5). The nuclei were stained with DAPI, and cells were viewed using confocal microscopy. Goat raised anti-GFP primary antibody with anti-goat IgG:FITC secondary antibody, and anti-goat IgG:FITC antibody alone (isotype control) were used as negative controls to confirm the specificity of anti-IFN-β antibody. IFN-β protein (FITC, green) was detectable intracellularly after H3N2 infection, Poly I:C stimulation and in un-treated media controls in the absence of cycloheximide. Poly I:C stimulation resulted in an elevated level of IFN-β protein inside pBECs compared to H3N2 and media. Cycloheximide pre-treatment in each of these conditions resulted in the inability to detect intracellular IFN-β in pBECs, suggesting that all IFN-β had been released into the supernatants as observed in Figure 4C.

The presence of intracellular IFN-β was also confirmed in differentiated, uninfected pBECs (Figure S3). Differentiated pBECs were stained with H&E stain for nucleus and alcian blue for mucus to indicate successful differentiation at air-liquid interface (Figure S3A). Differentiated pBECs showed similar sialic acid residue expression (Figure S3B) and the presence of intracellular IFN-β (Figure S3C) as that observed in non-differentiated cells.

Collectively these results provide further evidence for the presence of pre-formed constitutive IFN-β protein in pBECs, which can be released into supernatants upon host protein inhibition.

### Constitutive IFN-β release plays a critical role in suppressing influenza infection

**Figure 6:**
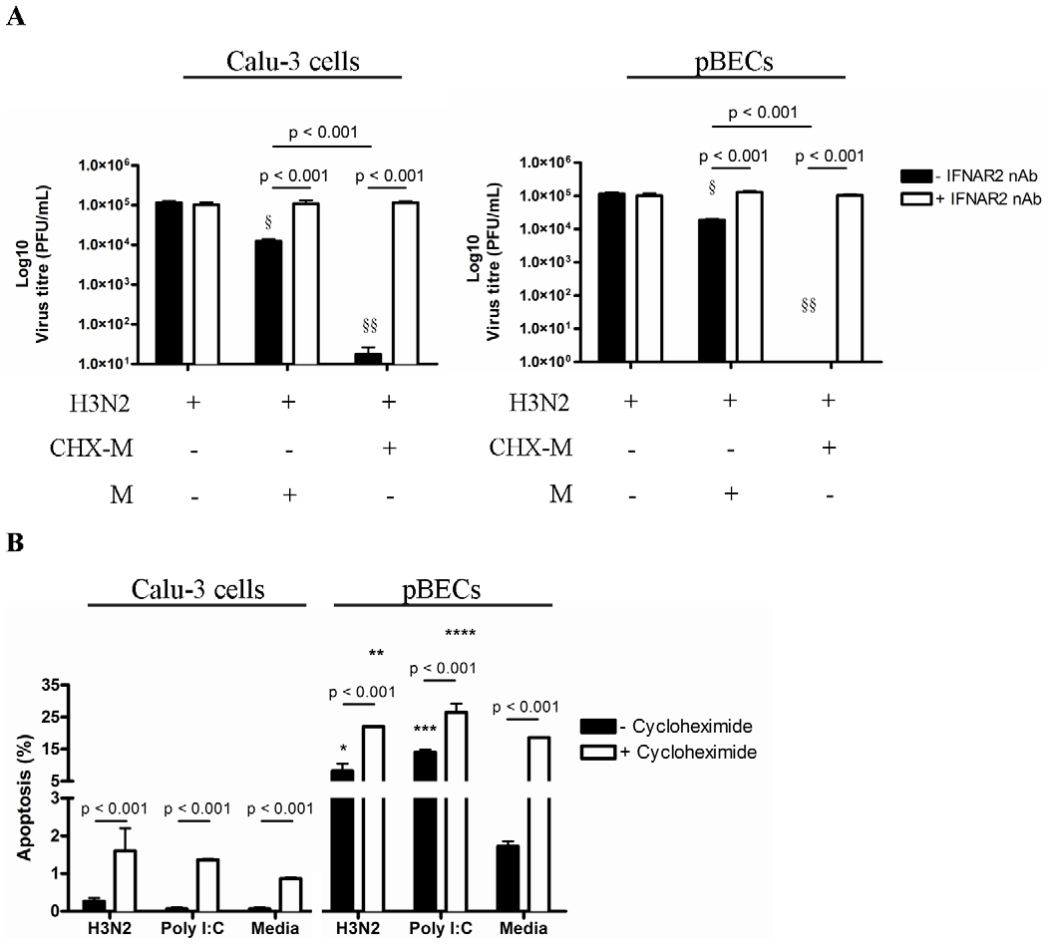
Anti-H3N2 influenza virus activity of constitutive IFN-β in limiting H3N2 plaque formation. (A) Supernatants from cycloheximide-treated un-infected Calu-3 cells and pBECs (CHX-M) was mixed with H3N2 influenza virus (1×10^5^ pfu/ml), and standard plaque assay was performed on MDCK cells with and without IFNAR2 neutralization. IFNAR2 nAb was added to MDCK cells 1 h before plaque assay was performed. (B) Apoptosis was measured by staining the cells with AxV and 7-AAD and analysis using flow cytometry 6 h after H3N2 and Poly I:C treatment. Apoptotic cells were AxV positive and 7-AAD negative. Results were derived from three independent experiments and are presented as mean ± standard error of the mean (SEM). * - **** indicates significant induction from media control. § and §§ indicates significant reduction compared to media control.

To determine the functional role of constitutive IFN-β during influenza infection, we assessed if enhanced IFN-β release was associated with increased antiviral activity in limiting influenza plaque formation. Supernatants of cycloheximide-treated (CHX-M) or untreated (M) Calu-3 cells and pBECs media control cells were mixed with H3N2 virus (1×10^5^ pfu/ml) and standard plaque assays were performed on MDCK cells. CHX-M dramatically suppressed H3N2 plaque formation compared to a partial but significant decrease induced by M (Figure 6A). In contrast, neither supernatant reduced viral titres when the IFN-β receptor IFNAR2 was blocked with neutralizing antibody (IFNAR2 nAb) on MDCK cells in the plaque assay. This demonstrates that the increased IFN-β protein in the cycloheximide treated cells effectively inhibited viral plaque formation.

Apoptosis has previously been known to limit viral replication [30], [31]. Thus we then assessed the role of IFN-β in host cell apoptosis during influenza infection (Figure 6B). Cycloheximide treatment alone caused a significant induction of apoptosis that progressed with time, especially in pBECs (data not shown). This effect was accelerated in pBECs when also infected with H3N2 or treated with Poly I:C (Figure 6B). In Calu-3 cells where viral replication occurred more effectively (Figure 1), there was relatively less apoptosis, though it still increased compared to control cells (Figure 6B).

**Figure 7:**
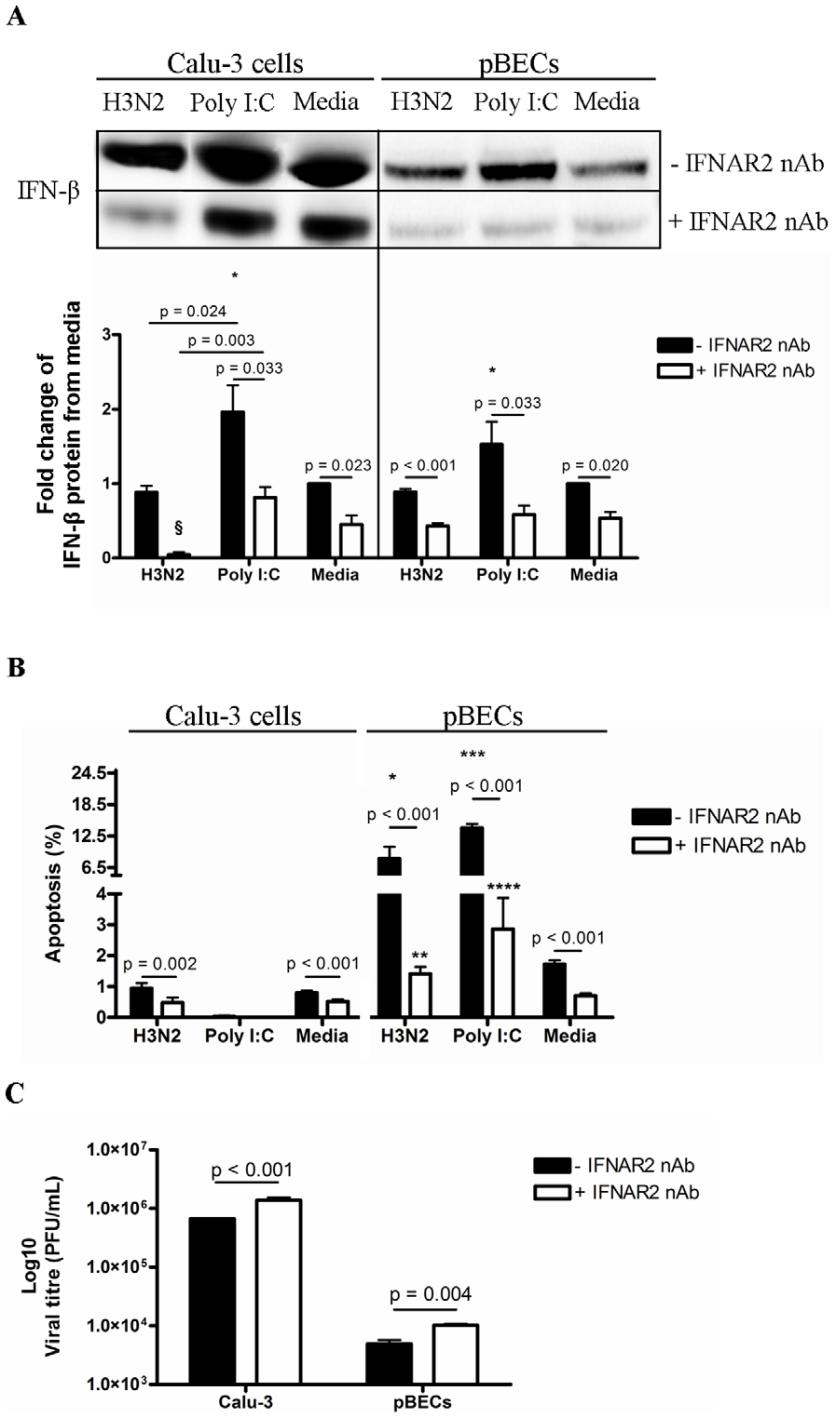
H3N2 influenza virus replication, IFN-β production and apoptosis in IFNAR2 neutralized Calu-3 cells and pBECs. IFNAR2 was blocked with IFNAR2 neutralizing antibody in Calu-3 cells and pBECs before H3N2 influenza virus infection or treatment with Poly I:C. (A) IFN-β production was assessed at 48 h after H3N2 and Poly I:C treatment by western blotting. (B) Apoptosis was measured at 6 h using flow cytometry. (C) Viral replication was measured by plaque assay after 48 h. Western blots of Calu-3 cells and pBECs were performed separately. Results were derived from three independent experiments and are presented as mean ± standard error of the mean (SEM). * - **** indicates significant induction from media control. § indicates a significant reduction compared to media control.

To further confirm the role of the constitutive IFN-β in influenza infection, we blocked the IFNAR2 during H3N2 infection with a neutralizing antibody (Figure 7). Blocking the IFN-β receptor substantially reduced IFN-β protein levels following infection, Poly I:C stimulation and in the media control (Figure 7A). The decrease in IFN-β was accompanied by reduced apoptosis during infection (Figure 7B). This led to significant increases in H3N2 titres compared to un-treated cells (Figure 7C).

Collectively these results suggest that while IFN-β production was inhibited by H3N2, the production and activity of constitutive IFN-β together with viral recognition was sufficient to trigger host cell apoptosis and suppress viral replication.

### Apoptosis is critical in limiting H3N2 replication

**Figure 8:**
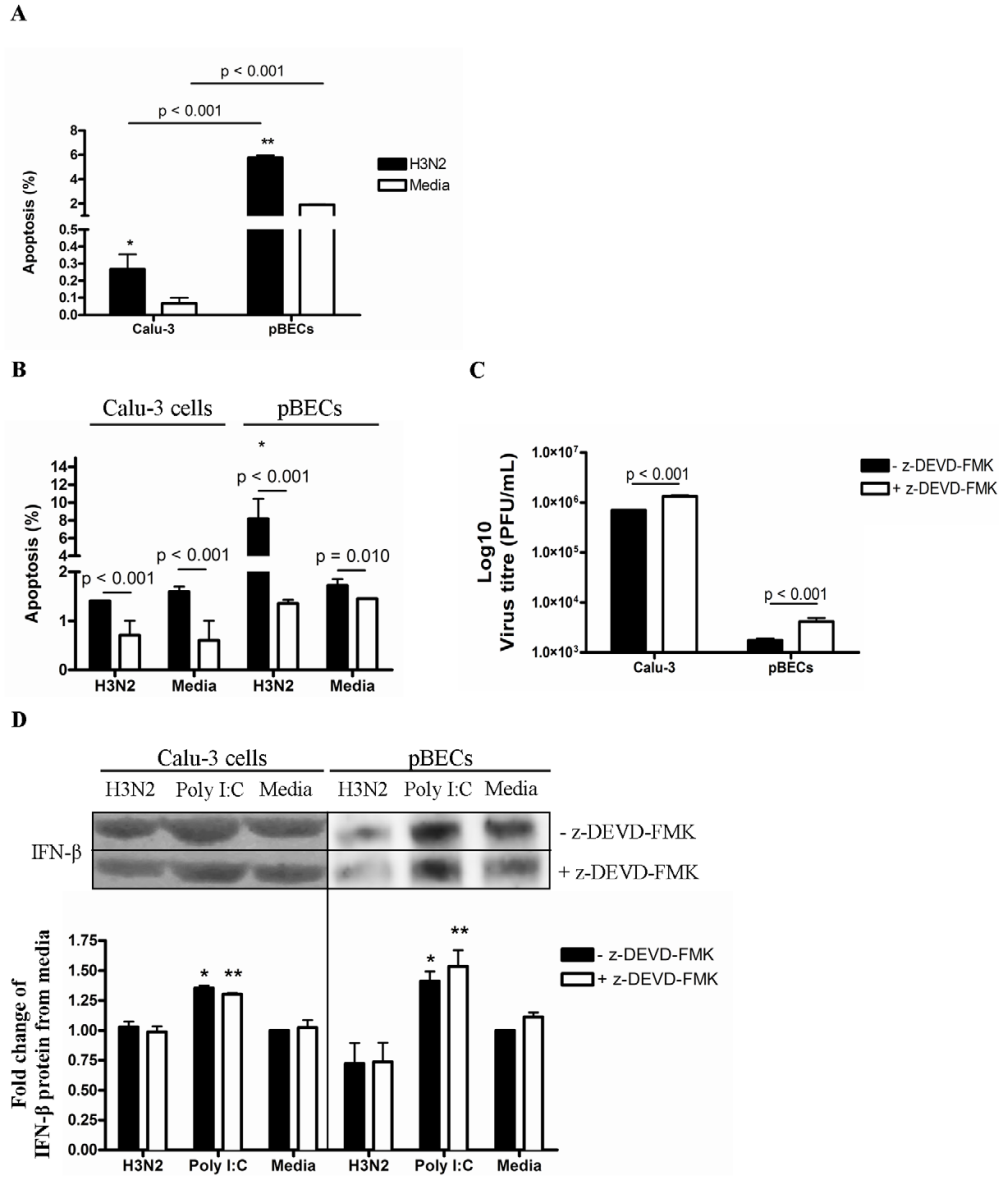
Effect of apoptosis on H3N2 replication in Calu-3 cells and pBECs. Calu-3 cells and pBECs were infected with H3N2 influenza virus or treated with Poly I:C. (A) Apoptosis was measured at 6 h after H3N2 and Poly I:C treatment by using flow cytometry. (B) z-DEVD-Fmk was administered 3 h before H3N2 and Poly I:C stimulation to similar cultures to inhibit apoptosis, and apoptosis was measured at 6 h. (C) Viral replication was measured by plaque assay after 48 h. (D) IFN-β production was assessed at 48 h after H3N2 and Poly I:C treatment by western blotting. Western blots of Calu-3 cells and pBECs were performed separately. Results were derived from three independent experiments and are presented as mean ± standard error of the mean (SEM). * and ** indicates significant induction from media control.

Calu-3 cells and pBECs had similar magnitudes of antiviral responses to infection, but supported very different levels of H3N2 replication. Therefore, we then further investigated the mechanisms of differential replication by measuring apoptosis levels during infection in Calu-3 cells and pBECs (Figure 8).

Increased viral titres in infected Calu-3 cells (Figure 1A) were accompanied by reduced levels of apoptosis compared to pBECs (Figure 8A). To determine if apoptosis directly impaired viral replication, the pan-caspase inhibitor z-DEVD-Fmk was used to inhibit apoptosis during infection. Treatment with the inhibitor reduced apoptosis during H3N2 infection (Figure 8B) and significantly increased viral replication in both cell types (Figure 8C), though IFN-β protein levels remained unchanged (Figure 8D).

This indicates that host cell apoptosis is crucial in limiting influenza viral replication, especially in pBECs, and is induced by the combination of infection and constitutive IFN-β release.

### H5N1 replicated to high titre with complete abolishment of antiviral responses and apoptosis in infected BECs

While H3N2 effectively inhibited inducible IFN-β to facilitate virus replication, replication was still limited through the effect of constitutive IFN-β release and host cell apoptosis. We then investigated whether the highly pathogenic H5N1 strain might have enhanced virulence through more potent inhibition of these host defences.

**Figure 9:**
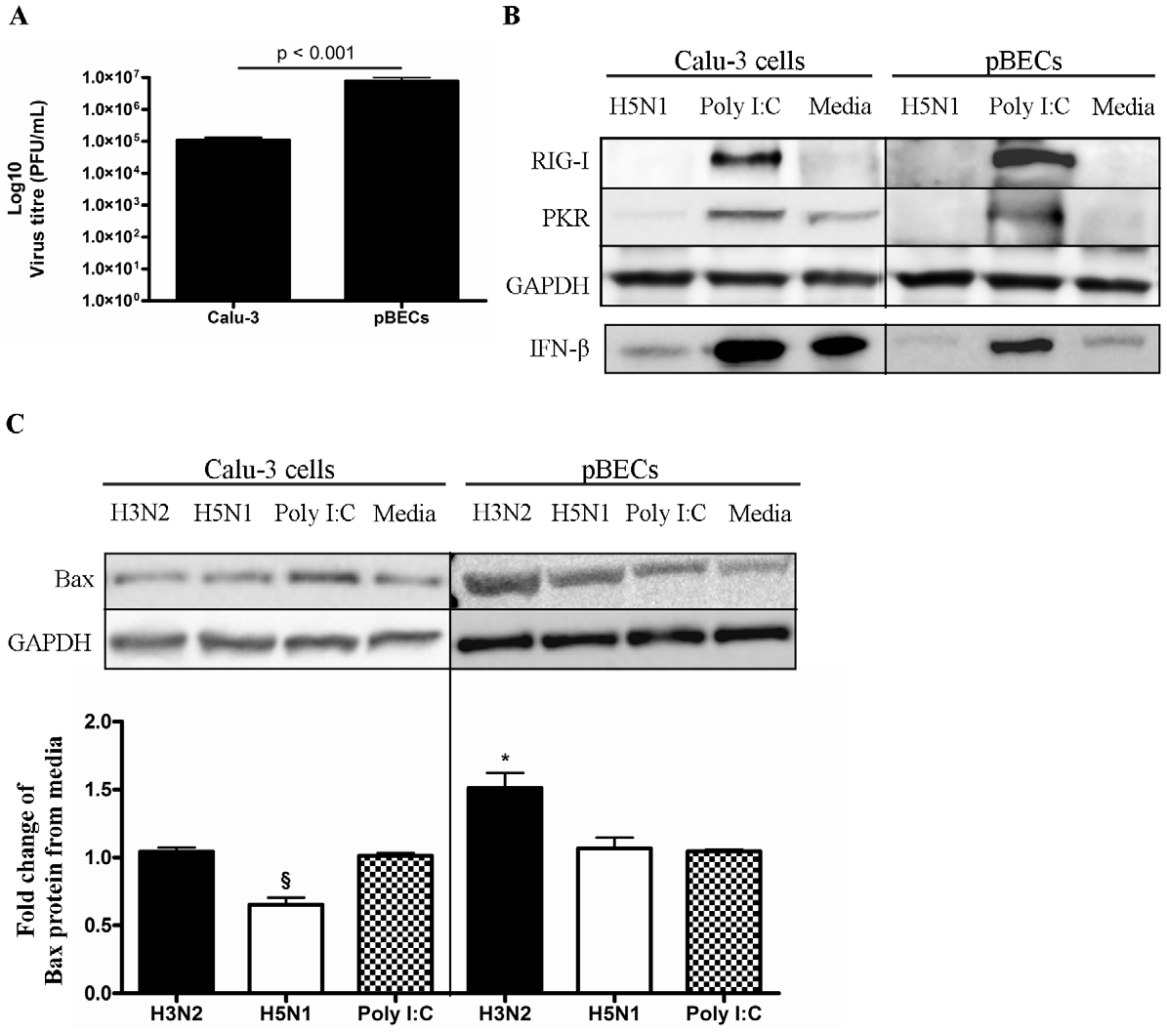
H5N1 influenza virus replication, type I IFN signalling and responses, and apoptosis in Calu-3 cells and pBECs. Calu-3 cells and pBECs were infected with H5N1 influenza virus at MOI of 0.005. (A) Viral replication was measured by plaque assay after 48 h. (B) RIG-I and IFN-β, and (C) Bax were assessed at 6 h after H3N2 and Poly I:C treatment by western blotting. Western blots of Calu-3 cells and pBECs were performed separately. Results were derived from three independent experiments and are presented as mean ± standard error of the mean (SEM). * and ** indicates significant induction from media control. § indicates a significant reduction compared to media control.

H5N1 infection of Calu-3 cells was initially performed at an MOI of 5, however this led to a complete destruction of the cell monolayer. Subsequently an MOI of 0.005 was found to be optimal in cell viability as this dose led to minimal cytopathic effect. Even at this much reduced dose H5N1 replicated more to a higher titre in both cell types (Figure 9A) compared to H3N2 infection (Figure 1A). This was despite the limited expression of SAα2,3Gal residues that are the preferred receptors for avian influenza (Figure 1B). H5N1 titre was significantly higher in pBECs than in Calu-3 cells (Figure 9A), in contrast to that observed with H3N2 infection (Figure 1A). Also in contrast to H3N2, H5N1 infection resulted in no induction of RIG-I, PKR, and IFN-β mRNA (data not shown) or protein (Figure 9B). As a result of constraints in working with H5N1 it was not possible to measure apoptosis using flow cytometry, therefore apoptosis was measured by assessing Bax protein expression (Figure 9C). In agreement with results using AxV/7AAD staining (Figure 8A), Bax was only induced above baseline levels in pBECs but not Calu-3 cells during H3N2 infection (Figure 9C). H5N1 infection did not up-regulate Bax expression in either cell type (Figure 9C).

Collectively these results indicate that H5N1 infection effectively inhibited both the inducible IFN-β response and the action of constitutive IFN-β on inducing apoptosis that enabled high levels of viral replication.

## Discussion

Here we demonstrate that antiviral response to pathogenic human and avian influenza infection in airway epithelial cells is critically dependent on constitutive IFN-β release and IFN-β signalling rather than the abundance of sialic acid residues or RIG-I-mediated pathways as previously thought. H3N2 infection was able to limit inducible IFN-β induction in BECs, however the constitutive release of IFN-β provided an effective innate immune response that limited viral replication in the infected BECs. In pBECs there was an early induction of RIG-I and a greater proportion of infected cells underwent apoptosis that was associated with reduced viral replication. However the RIG-I responses was not required to suppress viral replication. Importantly, the constitutive release of IFN-β played a critical role in triggering apoptosis and limiting viral replication in infected BECs. Furthermore, blocking the type I IFN receptor or apoptosis enhanced viral replication. In contrast infection of BECs with H5N1 resulted in inhibition of both inducible and constitutive IFN-β release, and impaired apoptosis that led to highly efficient viral replication, demonstrating the important role the innate immune response in determining early antiviral responses.

Calu-3 cells and pBECs are well characterized and accepted as a model of the proximal airway epithelium. Calu-3 cells and pBECs have been used to assess immune responses to other viral infection such as rhinovirus, by our group and others [32]–[35]. We have also previously characterized the response of Calu-3 cells to influenza infection, which was also confirmed in pBECs in our previous study [36]. However this study demonstrated important differences in antiviral responses to infection and susceptibility to influenza infection. Lower levels of SAα2,6Gal linked glycoprotein expression were observed on Calu-3 cells compared to pBECs, however H3N2 replication was greater in Calu-3 cells. In addition, avian H5N1 strain, although used at a significantly lower dose, replicated to a even higher extent in Calu-3 cells and pBECs, despite of the low levels of SAα2,3Gal residues. These observations are in accordance with our previous work that demonstrated similar HA levels immediately after human and avian influenza viral infection in Calu-3 cells and pBECs irrespective of these sialic acid residues [26]. These sialic acid-bearing glycoproteins therefore may only have a minor role in susceptibility, and that post-endocytosis antiviral responses are more important determinants of influenza replication.

Antiviral responses are critically important in suppressing viral spread early in infection. In our study pBECs were more resistant to H3N2 infection due to higher levels of apoptosis after infection, which led to reduced viral replication. In contrast Calu-3 cells did not readily undergo apoptosis, which resulted in more efficient replication. The reason for this difference in apoptosis is probably due to the nature of immortalized cell lines, and results from pBECs provide a better insight into the natural human host cell response to influenza infection.

Primary BECs showed early and late increases in RIG-I expression after H3N2 infection. While numerous studies demonstrated the importance of RIG-I in influenza RNA recognition and subsequent signal transduction to induce IFNs [17], [37]–[42], in this study suppressing RIG-I affected neither IFN-β protein expression nor H3N2 replication efficiency in pBECs. This suggests that while RIG-I is critical in viral RNA recognition, RIG-I-signalling may not be important in influenza infection as its signalling pathway is interfered by the influenza NS1 protein [36], [43], and other factors are critical in controlling viral replication. The lack of IFN-β induction after H3N2 infection is known to result from the suppression by the influenza NS1 protein. We have previously shown that the NS1 protein of the H3N2 strain was more potent in inhibiting the antiviral type I IFN response than that of a low pathogenic H11N9 avian influenza virus [26]. The host binding targets of NS1 include IRF3 phosphorylation [44], TRIM25 [45], NK-kB [46]–[48], and cellular mRNA translation processes [49]. This results in impaired innate immune responses in infected host cells.

Despite this inhibition of inducible type I IFN in BECs, we still observed a selective induction of RIG-I and PKR protein late in H3N2 infection, and the constitutive release of IFN-β played a pivotal role in this late antiviral signalling. Cycloheximide was used as a general inhibitor of host protein synthesis to determine if the IFN-β was induced in response to infection or was pre-existing. Treatment with cycloheximide resulted in complete loss of GAPDH protein. GAPDH has been shown to be degraded in the presence of oxidative stress in eukaryotic cells [50], and cycloheximide has significant toxic side effect that enhances intracellular oxidative stress, that together with the inhibition of host protein synthesis resulted in this loss. Cycloheximide also resulted in the release of the intracellular pool of pre-formed IFN-β into infection supernatants, which was important in up-regulating apoptosis and limit viral replication, before it had any effect on cell viability. The constitutive IFN-β was functionally active as the enhanced IFN-β in cycloheximide treated cells effectively limited H3N2 replication, and inhibition of IFNAR2 markedly reduced IFN-β-induced apoptosis and increased viral replication. The antiviral effect of type I IFN in reducing viral replication is well established [51], and we have previously shown that exogenous IFN-β was able to reduce viral replication of H3N2 and a low pathogenic avian H11N9 strain [36]. We cannot rule out that type III IFNs are also involved in limiting influenza viral replication. Type III IFNs are induced and functions in a similar fashion to type I IFNs that amplify antiviral ISGs, and while both IFN-β and IFN-λ1 were found to be critical in reducing viral replication, we have also shown that IFN-λ1 was not induced upon H3N2 infection [36]. Influenza, as well as many other RNA viruses such as herpes simplex virus are known to preferentially and effectively inhibit host cell protein synthesis and promote viral protein synthesis and replication [52]. Our results suggest that the inhibition of protein synthesis by the virus may acts as a trigger for the release of pre-formed IFN-β and apoptosis in the infected host cells and this release appeared to be RIG-I- and apoptosis-independent. Cycloheximide has been shown to induce apoptosis [53], [54], and while elevated apoptosis level with cycloheximide treatment correlated with increased IFN-β release, it is unlikely that the constitutive IFN-β release was dependent on apoptosis. The characteristics of apoptosis including cell shrinkage with maintained membrane integrity without leakage of internal materials suggest the constitutive IFN-β release involves novel signalling pathways. A similar response has also been observed in cells infected with Newcastle Disease virus (NDV). Cells treated with cycloheximide during NDV infection released greater amounts of IFN-β, which was thought to occur through enhanced stability of IFN mRNA [55]. This, however, does not explain the constitutive release of IFN-β protein when host protein synthesis was blocked at the protein translational level by cycloheximide. While the exact mechanism by which IFN-β protein is released when protein synthesis is blocked is unclear, this constitutively released IFN-β is functionally more important in limiting viral replication *via* IFN-β-IFNAR2 signalling than the initial RIG-I initiated signalling.

The constitutive release of IFN-β demonstrated here has been reported previously *in vitro* and *in vivo* [56]–[60], and was hypothesized to play a role in the priming and enhancement of IFN response, as proposed in a “revving-up model” [59], [61]. A constitutive weak signal induced by IFN-β-IFNAR2 signalling allows epithelial cells to elicit a more robust response toward viral infection, while in the absence of this signal epithelial cells become hypo-responsive to this stimulus. Despite the critical role of constitutive IFN-β in H3N2 infection, H5N1 inhibited all antiviral signalling more effectively in infected cells, including signalling induced by constitutive IFN-β. This may have occurred as a result of the potent suppressive properties of the H5N1 NS1 protein [23], [25], [27]–[29], [62]–[65], leading to highly efficient viral replication in these cells.

Collectively our results demonstrate that the constitutive type I IFN response of infected BECs is important in determining the level of influenza replication. Influenza viral replication is not dependent on the sialic acid residue-bearing glycoproteins, and the antiviral responses have an important role in limiting virus replication, particularly the effect of constitutive release of IFN-β during infection. In the early phase of infection epithelial cells have limited ability to produce IFN-β due to effective influenza NS1 protein. However constitutive IFN-β response counteracts some of the suppressive effects of the influenza virus, via IFNAR2 signalling to induce expression of ISGs including RIG-I and PKR. These ISGs are important in establishing an antiviral state in BECs as they are the effector antiviral proteins that inhibit viral mRNA translation, inducing apoptosis and amplify the entire IFN response.

## Materials and Methods

### Influenza virus

Human influenza A/Wellington/43/2006 (H3N2) strain and A/Vietnam/1203/04 (H5N1) was obtained from the WHO Collaborating Centre for Reference and Research on Influenza (Vic, Australia). Influenza viruses were propagated and virus titres determined by plaque assays on Madin-Darby canine kidney (MDCK) cells (American Type Culture Collection (ATCC), USA) [66]. Ultra-violet (UV) inactivation of live viruses was achieved by placing live viruses directly under UV lamp (254 nm) for 4 h. Successful inactivation was confirmed by plaque assays. All work with H5N1 was performed in the level 4 containment facility at The Commonwealth Scientific and Industrial Research Organization (CSIRO) - Australian Animal Health laboratory, Geelong, Victoria, Australia.

### Cell culture and viral infection

Calu-3 and MDCK cells (ATCC) were maintained in minimum essential media supplemented with 10% fetal bovine serum and Dulbecco's modified Eagle's media with 5% fetal bovine serum, respectively [26]. Human pBECs were obtained from healthy individuals by endobronchial brushing during fibre-optic bronchoscopy [67]. Subjects had no history of smoking or lung disease and had normal lung function. All subjects gave written consent. pBEC were cultured as described [26], [35]. For differentiation of pBECs, cells were grown on transwells (Corning) at air-liquid interface, with basolateral media changed every second day at 37°C/5% CO_2_ [68].

H3N2 and H5N1 were diluted in the appropriate serum free media and added to cells at multiplicity of infection (MOI) of 5 and of 0.005, respectively. After 1 h of incubation, the inocula were removed and replaced with serum-free media. Cells were treated with exogenous polyinosinic∶polycytidylic acid (Poly I:C, 100 µg/ml, Sigma-Aldrich), a known agonist of RIG-I and IFN responses, as a positive control [36], [69]. Cycloheximide (100 ug/ml, Sigma-Aldrich) was used to inhibit protein synthesis by pre-treatment of cells (30 min, 37°C, 5% CO_2_) and was added to media after virus inoculation. Caspases inhibitor Z-DEVD-fmk (Calbiochem, USA) of 50 µM was used to pre-treat the cells for 3 h before infection and was added after 1 hr virus inoculation. In experiments involving the blockade of IFNAR2, cells were incubated with 1 µg/ml of mouse monoclonal neutralizing antibody to IFNAR2 (CD118, PBL Laboratories) for 1 h prior to virus inoculation.

### Antiviral assay

Supernatants from cycloheximide treated and untreated uninfected media control cells were standardized to 100 µg and added to H3N2 virus at 1×10^5^ plaque forming unit (pfu)/ml and standard plaque assay was performed on MDCK cells [66].

### RNA interference

siRNA to RIG-I (Applied Biosystems) was complexed with siPORT NeoFX transfection agent (Applied Biosystem) and diluted to 10 nM with OPTI-MEM-I (Invitrogen). The siRNA complex was added to the cells and induced >60% knockdown after 24 hr. H3N2 infection was performed at this point.

### Flow cytometry

FITC conjugated *Sambucus niger* agglutinin (SNA) and *Maackia amurensis* (MAA) II lectins (Vector Laboratories) were used to identify SAα2,6Gal and SAα2,3Gal residues, respectively. BECs were stained with FITC-SNA at 10 µg/ml and incubated at 4°C for 1 h. The cells were then analysed using flow cytometry (FACSCanto II, Becton Dickinson) and results were expressed as median fluorescence intensity [26].

### Apoptosis

Apoptosis was measured using PE Annexin V Apoptosis Detection kit I (Becton Dickinson) according to manufacturer's instruction. Cells were harvested and stained with annexin V-PE (AxV) stain and vital dye 7-amino-actinomycin (7-AAD) and then analyzed using a FACSCanto II (Becton Dickinson) and FACSDiva software. Apoptotic cells were stained AxV positive/7-AAD negative and expressed as percentage of total analyzed cells.

### RT-qPCR

RNA was extracted from infected Calu-3 cells using RNeasy Mini Kits (Qiagen) according to the manufacturer's instructions. RNA (1 µg) was reverse transcribed to cDNA and was used for RT-qPCR assays (Applied Biosystem). Ribosomal RNA (18S) was used as the reference gene. The cycle threshold (Ct) value obtained was normalized to that of the 18S gene, and expressed as fold induction over the media control.

### Immunoblotting

Calu-3 cells and pBECs were lysed in RIPA buffer, and all proteins were standardized to 10 µg and were resolved by SDS-PAGE, and transferred onto nitrocellulose membranes for detection of IFN-β in supernatants, RIG-I, PKR, and Bax in cell lysates. GAPDH was detected as a loading control for proteins in cell lysates. Protein estimation was determined by densitometry and the values were expressed as protein/GAPDH ratio and presented as fold induction from media control. As there is no loading control available for secreted proteins, densitometric values for IFN-β was presented as fold induction from media control.

### Immunofluorescence and confocal microscopy

Calu-3 cells and pBECs were fixed in 4% paraformaldehyde/4% sucrose, permeabilized with 0.5% Triton X-100/PBS, and subjected to confocal microscopy. Cells were stained with goat raised anti-human IFN-β primary antibody (1 µg/ml, Abcam) and anti-goat secondary IgG:FITC (5 ng/ml, Abcam), and counter-stained and mounted with ProLong Gold antifade reagent with DAPI (Invitrogen). Goat raised anti-green fluorescent protein (GFP) primary antibody (1 µg/ml, Abcam) with secondary anti-goat IgG:FITC (5 ng/ml), and anti-goat IgG:FITC (5 ng/ml) alone were used as negative staining controls. The differentiated pBECs was sectioned, and stained with alcian blue for mucus and H&E stain for the nucleus to confirm cell morphology. Sialic acid residues (FITC conjugated SNA and APC conjugated MAA II lectins, Vector Laboratories) and the presence of intracellular IFN-β was also stained in differentiated pBECs. The cells were viewed with a confocal laser scanning microscope (Olympus FluoView 1000, Olympus). Images were digitally recorded and analysed by FV10-ASW (Olympus).

### Antibodies

All antibodies used in this study are commercially available and have been validated by the source companies.

### Data analysis

Data were expressed as mean ± standard error of mean (SEM). All data presented in the results were normally distributed, and statistical analysis was performed using student's two-tailed *t* test. A p-value of <0.05 was considered significant. The study was approved by The University of Newcastle Human Research Ethics Committee.

## Supporting Information

Figure S1 **Induction of type I IFN gene expression in response to H3N2 influenza virus infection in Calu-3 cells.** Calu-3 cells were infected with H3N2 influenza virus or treated with Poly I:C. (A) RIG-I, (B) IFN-β and (C) PKR mRNA was measured by RT-qPCR at 6, 12, 24, 48 and 72 h after infection. UV-inactivated virus was no different to the media control (data not shown). Results were derived from three independent experiments and are presented as mean ± standard error of the mean (SEM).
(TIFF)

Figure S2 **Induction of type I IFN gene expression in response to H3N2 influenza virus infection in pBECs.** pBECs were infected with H3N2 influenza virus or treated with Poly I:C. (A) RIG-I, (B) IFN-β and (C) PKR mRNA was measured by RT-qPCR at 6, 12, 24, 48 and 72 h after infection. Results were derived from three independent experiments and are presented as mean ± standard error of the mean (SEM).
(TIFF)

Figure S3 **Cell morphology, SAα2,6Gal and SAα2,3Gal residue expression, and intracellular IFN-β in differentiated pBECs.** pBECs were cultured at an air-liquid interface. (A) The differentiated phenotype was assessed by H&E staining for nuclei and alcian blue staining for mucus. (B) SAα2,6Gal and SAα2,3Gal residues were stained with FITC-SNA and APC-MAL-II, respectively, and assessed using confocal microscopy. (C) IFN-β was also stained with goat raised anti-IFN-β with anti-boat IgG:FITC and assessed with confocal microscopy. Results were derived from three independent experiments.
(TIFF)
