# Supplementary figures and images for: Critical Role of Constitutive Type I Interferon Response in Bronchial Epithelial Cell to Influenza Infection
Source: PLoS One. 2012 Mar 2;7(3):e32947. doi: 10.1371/journal.pone.0032947 (PMC3292582; doi:10.1371/journal.pone.0032947)

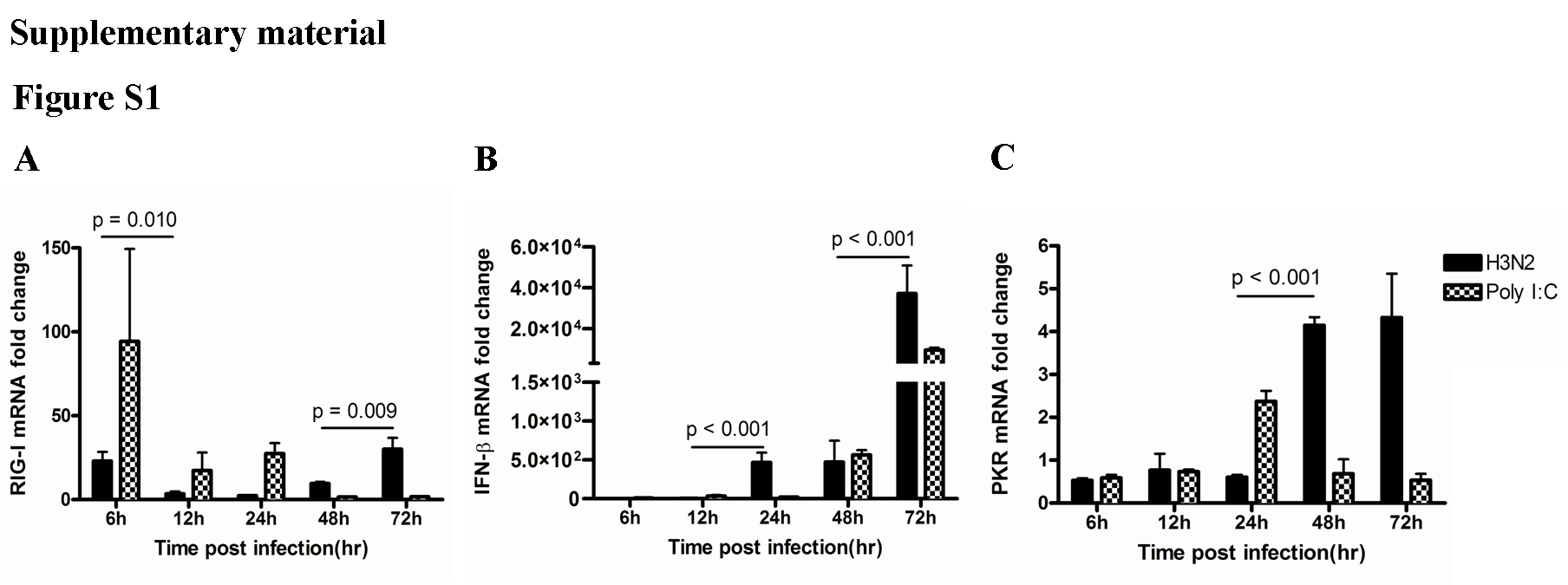

Supplement: Figure S1 — Induction of type I IFN gene expression in response to H3N2 influenza virus infection in Calu-3 cells. Calu-3 cells were infected with H3N2 influenza virus or treated with Poly I:C. (A) RIG-I, (B) IFN-β and (C) PKR mRNA was measured by RT-qPCR at 6, 12, 24, 48 and 72 h after infection. UV-inactivated virus was no different to the media control (data not shown). Results were derived from three independent experiments and are presented as mean ± standard error of the mean (SEM). (TIFF) [file pone.0032947.s001.tiff]

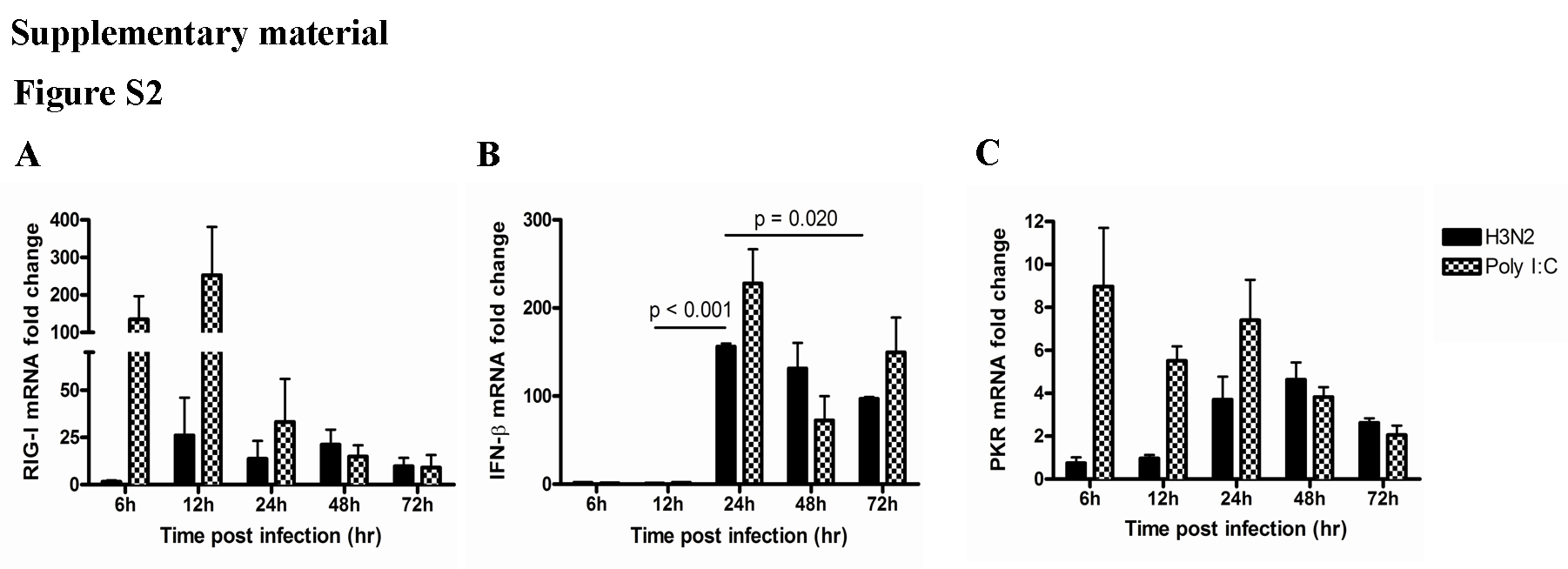

Supplement: Figure S2 — Induction of type I IFN gene expression in response to H3N2 influenza virus infection in pBECs. pBECs were infected with H3N2 influenza virus or treated with Poly I:C. (A) RIG-I, (B) IFN-β and (C) PKR mRNA was measured by RT-qPCR at 6, 12, 24, 48 and 72 h after infection. Results were derived from three independent experiments and are presented as mean ± standard error of the mean (SEM). (TIFF) [file pone.0032947.s002.tiff]

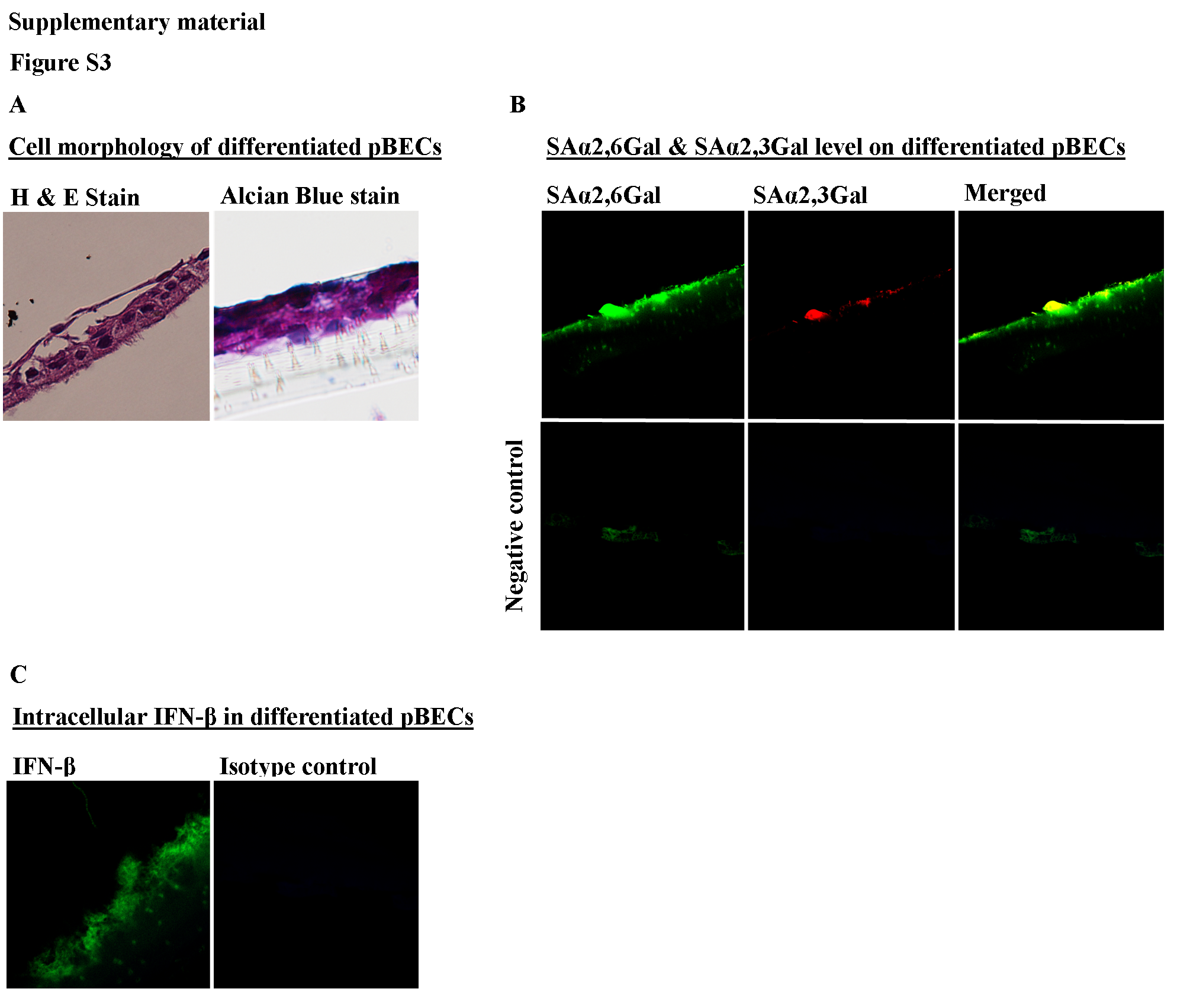

Supplement: Figure S3 — Cell morphology, SAα2,6Gal and SAα2,3Gal residue expression, and intracellular IFN-β in differentiated pBECs. pBECs were cultured at an air-liquid interface. (A) The differentiated phenotype was assessed by H&E staining for nuclei and alcian blue staining for mucus. (B) SAα2,6Gal and SAα2,3Gal residues were stained with FITC-SNA and APC-MAL-II, respectively, and assessed using confocal microscopy. (C) IFN-β was also stained with goat raised anti-IFN-β with anti-boat IgG:FITC and assessed with confocal microscopy. Results were derived from three independent experiments. (TIFF) [file pone.0032947.s003.tiff]
